# An Overview of Recent Advances of Resilient Consensus for Multiagent Systems under Attacks

**DOI:** 10.1155/2022/6732343

**Published:** 2022-08-02

**Authors:** Muhammad Muzamil Aslam, Zahoor Ahmed, Liping Du, Muhammad Zohaib Hassan, Sajid Ali, Muhammad Nasir

**Affiliations:** ^1^School of Computer and Communication Engineering, University of Science and Technology, Beijing 100083, China; ^2^Department of Electronics and Communication Engineering, University of Science and Technology China (USTC), Hefei, China; ^3^Department of Information Sciences, University of Education Multan Campus, Lahore, Pakistan; ^4^Department of Automation, Shanghai Jiaotong University, Shanghai 200240, China; ^5^Department of Computer Engineering, The University of Lahore, Lahore, Pakistan

## Abstract

Consensus control of multiagent systems (MASs) has been one of the most extensive research topics in the field of robotics and automation. The information sharing among the agents in the MASs depends upon the communication network because the interaction of agents may affect the consensus performance of the agents in a communication network. An unexpected fault and attack may occur on one agent and can propagate through the communication network into other agents. Thus, this may cause severe degradation of the whole MASs. In this paper, we first discussed MAS technologies. After that available technologies for the modeling of attacks and fundamental issues due to attacks on MAS attacks were discussed. We also introduced cooperative attack methodologies and model-based attack methodology. Objective of this article is to provide comprehensive study on recent advances in consensus control of MASs under attacks covering the published results until 2021. This survey presents different kinds of attacks, their estimation and detection, and resilient control against attacks. At the end, the survey accomplishes some potential recommendations for future direction to solve the key issues and challenges reported for secure consensus control of MASs.

## 1. Introduction

With advancement of communication and computer technologies, coordination control of MASs has got a lot of attention of researchers in different areas of engineering due to its broad applications in order to attain craving physical performances [1, 2]. There is speedy increment in progress of MASs because of improvement in communication, computing, and relevant technologies. MASs are also known as integration of communications, computations, physical processes, and controls which can play a key role in infrastructure [3, 4]. Cyber threats posture an actual and increasing problem, and to date, many countries struggle to counter them have lagged. However, capability to protect in contradiction of an attack or invasion must be upheld, and any country would be well served by discouraging its opponents from acting in the first place, at least when it comes to the most serious actions, namely, cyber warfare. There is vital role of cyber security in the era of technology also is biggest challenge to secure information in these days of technology. The first idea regarding cyber security in our mind is “cybercrime, cyber threat, or cyberattack”; those are increasing day by day. Several private and public sectors are taking various measures to secure such crimes, attacks, or threats.

**Figure 1:**
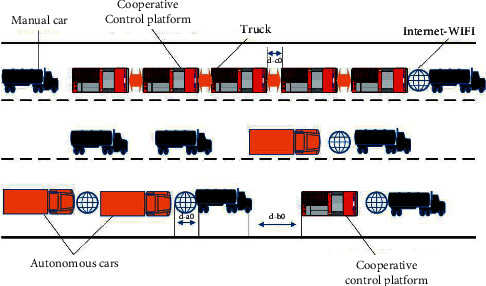
Intelligent transport system in coordination.

Due to variety of applications [4], MASs have become research interesting area. Despite usefulness, MASs have a lot of security risks because of the interconnections of various components and technologies. Since MASs are susceptible to spiteful threats and may result in the ailment of social life or loss of economic benefits. So, attack issues need considerable attention in the practical systems. In a smart transport system, an automatic controlled vehicle network can be a useful solution for an efficient and secure transport system and assists in problem-solving such as environmental protection, energy conservation, road congestion, and road accident. In contrast to the already present single-agent system, MASs are upgraded and scalable for enhancing energy efficiency and durability because they have the built-in ability for cooperative learning through the automatic decision. Recently, issues of MASs and their analysis have been well discussed in [5] but security issues of MASs have not been studied in the article and these systems are unsafe from cyberattacks and physical faults. So, we need to adjust, wait, or abandon at the time of system failure or attack [6]. MASs trust and access control issues have been highlighted. The researchers in [7, 8] highlighted MAS fault tolerant control procedure, and basic study was on reconfiguration way of topology. A very low-level attack or fault on an agent can disturb the system function and can cause damage to the system badly. Researchers have done wide research on cyber security and physical security in which detection and diagnosis, fault estimation problem, secure consensus, attack detection, and fault tolerant control were studied, and such research provides some basic methodologies and systematic suggestions for effective betterment of MASs security In Figure 1, there is framework of the intelligent transport system. Various kinds of attacks are present possible on the MASs such as man-in-middle attacks, deception attacks, cross-site scripting attacks, denial-of-service attacks, drive-by attacks, eavesdropping attacks, malware attacks, password attacks, phishing attacks, and SQL injection attacks.

In several fields of human activities, MASs have gained more attention, particularly the places where we need physical equipment, processes for control, and cooperating with the system and human, e.g., building automation networks, smart grids, and water sewerage plants. The progressing technologies, e.g., Industrial Internet or Industry 4.0 [9], are key points of MAS's importance; this idea transition will involve increasing autonomy, automation, and fulfilling fresh arrival of the production. The main motivation which is forcing the development of MASs is the conjunction of physical process and computational aptitudes, and components of the process happening in the physical surrounding are requirements. MAS scaling, e.g., large and small, is differentiated by the number of components involved [5].

These MASs are widely and geographically dispersed, heterogeneous, federated, and the critical system of life in which actuators and sensors are embedded networked for control physical world, monitoring, and sensing. There is no doubt that reserved scheduling via different own network or shared network plays a key role in the working of MAS. The selection of actuator or sensor for best performance of a specific action is an important decision, and also, proper management of actions is also important. Because of limited technology or physical constraints, there is risk of data transmission among network components, sensors, and actuators without any specific security instructions. In one point of view, interconnection of large scale of network components puts it in more complexity to save it from innate physical liabilities there. In another point of view, cyber integration generally sets up an underscore on protection and suppleness against threats and unseen from cyberspace [10, 11]. Hence, several new challenges are known to general or traditional control, software theory, and communication [12, 13].

The susceptibility of MASs in cyber parts permits attacks into the system in nonproductive and silent approach, e.g., insertion of some virus or worm in network component computerization or layers that should have medium access, could cause coordination packets distraction. In addition, there can be prohibited access of attacker to the monitoring centers and gaining decryption key in gain of normal operation [3]. It is clear that when there is no strict security protection, the attacker can either freely harm system dynamic or bring any agitation. For solution of optimization problem in selection of input or output that highlights attacks with least detect ability and maximum impact; there is a survey [14].

MAS consensus problem has been widely addressed [15, 16] with time delay. In MASs, we can found two types of delay, communication time delay (CTD) and input time delay (ITD). Here, CTD is linked between two agents interconnected time while ITD is related to data processing time. ITD may occur when controllers, actuators, or other components are linked to the network, while because of CTD, each agent may get information from a very near agent. CTD has been used in most real used applications such as computational and PWM delays in an LCL type grid-linked invertor and in controller area network bus of the distributed control network, induces delay. Here, we would like to discuss consensus problem of continuous time linear homogeneous MAS with CTD and ITD. ITD is considered multiple input time delays while CTD is single time delay. MAS consensus [14] problem has been widely studied from several studies, e.g., transportation control, coordinated defense system [17], power systems, and smart grids [18]. Consensus problem target is used to design a control law so that all agents can reach to a single point [14]. However, several present results on consensus problem were supposed as an ideal condition for investigation, e.g., secure network communication in between agents and unlimited resources network. In reality, there is presence of several impulsive issues, prime to a high security predicament and having influence on communication topology [14]. Nasty agents can grab this failing to interject communication between agents and disturb overall system stability in the multiagent network, while we can divide cyberattacks into the following cases on MASs, first is when there is an attack of nasty agent on agent and second is when nasty agent attacks to destroy the communication medium. In both cases, the communication graph is detached.

There is no high-level protection against future MAS threats or attacks. There are several types of key challenges regarding MAS security, and these threats may be from the system inside such as sudden failure of the system, mobile networks security risks regarding human health, communication protocol weakness in smart grids, and limitations of physical systems. There is increasing requirement in new control system accuracy and reliability of each component. Any type of failure or fault of a system or component may lead to system performance degradation or reason for system instability or dramatic change in system operation [19].

Because communication networks consist of interacting agents which require safety and reliability to gain cooperative control and given challenges which are absent in single-agent systems such as MAS interconnection properties confuse the fault detection and identification and make worldwide and exact fault information complicated to accommodate and gain. Faults can vary both network and agent behavior suddenly. Single-agent fault can effect throughout the communication network. There may occur multiple faults at various time intervals and places. However, such challenging problems can be studied in proper form. Multiple agent composition configuration can give better termination than a single-agent system. Other than this, throughout failure of component or an agent that is not possible to adjust for the single-agent system may be effectively controlled by use of cooperation in between network connection and agents.

**Figure 2:**
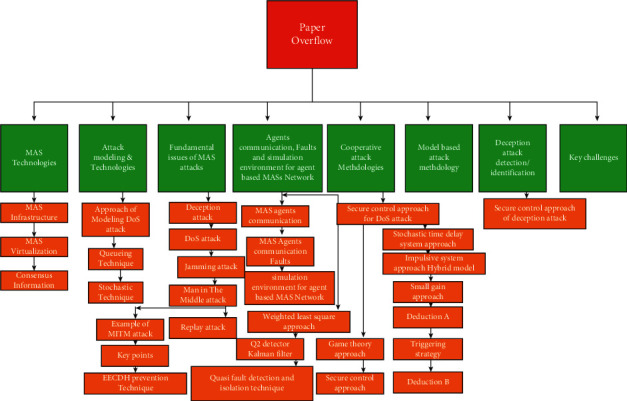
Paper flowchart.

Inspired by the above study, practical and theoretical importance is given to review and classify some impressive MAS attacks (Figure 5) and working (Figure 6) to give a complete survey, Figure 10 describes secure and control approaches of DoS attack, and Figure 2 shows present work and key challenges required for study. Furthermore, it is of both theoretical and practical significance to present a study for safety of MASs at one platform and also to provide comprehensive survey of recent advances of resilient consensus for MAS under attacks. To see latest MAS complexity, security problems and system security are highly important. Threats can be physical, cyber, or containing both sides of MAS, and it needs a composite method for mitigation and identification of safety weaknesses. In present research, the purpose was to study weakness, challenges, mitigation schemes, attack types supposing scalability complexity of MASs, and distributive and security and safety challenges. The remainder of the paper is organized as follows: in Section 2, we studied MAS technologies; in Section 3, we studied attack modeling and methodology; in Section 4, we briefly discussed fundamental issues of MASs; in Section 5, we discussed problem formation; in Section 6, there is comprehensive study on cooperative attack methodologies; in Section 7, there is discussion on model-based attack methodology, in Section 8, deception attack detection or identification has been studied; and in Section 9, there are briefly discussed key challenges. Similarly, Figure 2 represents flowchart of paper.  Table 1 explains all notations used in the manuscript.

## 2. MAS Supporting Technologies

Here, we study key technological improvements which are planned as well as links in between MAS and other basics which are studied to encourage technological developing chain that commanded the emergence of MASs. In several works, the concept such as smart object, embedded system, ubiquitous computing, smart environment, and sensor networks plays a key role in development of MASs. In case of time line, embedded systems are ancestors of all given knowledge appeared in the past and beginning fresh development in the field of microelectronics and replying critical problems such as remote control and automation. There is predefined functionality in embedded systems traversing across single or multiplied functions that are not easy to reprogram by the last user. The basic purpose of embedded systems was to control, design, and operate physical world process. Though, in beginning, embedded systems were closely compared to MAS or IoT, they were restricted by physical control function with the cyber space layer.

**Figure 3:**
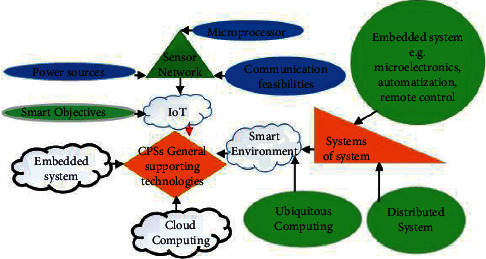
General technologies supporting MASs.

**Figure 4:**
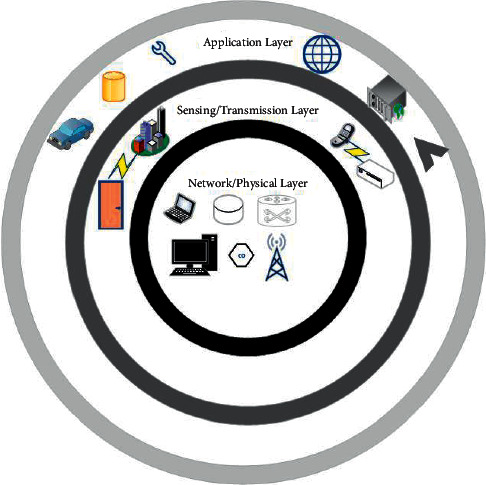
MAS infrastructure.

Figure 3 represents detailed general technologies supporting MASs. With the progress of technology and requirement to manage and control complicated systems, importance of the embedded system was clear and then there was an idea of the network embedded system. An important reason lacking down the availability of MAS was transition from one system to a connected system with more complexity. In view of this concept, sensor networks (SNs) strongly impact on the latest MAS. Actually, for information gathering, the SN contains a number of sensors deployed in specific place/areas. Presence of the SN eased improvements of smart objects, e.g., actuator or sensors containing microprocessor, power sources, and communication services. Thus, the SN cannot be supposed as freely unit, but as part of complicated systems. Fast development in modern computing systems containing MAS gave idea of ubiquitous computing. In addition, concept of computer system integration with daily activities enables them “unseen” for the last users. In some views, ubiquitous computing overlaps with other ideas, ambient intelligence, pervasive computing, and IoT. An important role of IoT is the establishment of capabilities for the last user to enhance present forms of everyday devices and improvement of private facilities by use of joined ubiquitous devices. It shows transition from the embedded system from predefined functionality to IoT and ubiquitous computing where an important need is adaptability and nimbleness. More and more improvements of computing systems run to presences of IoT and MAS. Actually, MAS is bit far from paradigm in which functional technologies are detached from information technologies to prototype where computational and physical elements are integrated. Although IoT has no specific definition, in some cases, IoT has been described as global infrastructure containing standard protocols and communication technologies that avail facilities given by “things” to diverse applications. In another case, IoT is a general term presenting the scenario types where smart objects are deployed, enabling worldwide communication through Internet or technologies. Hence, the concept of IoT is to represent the infrastructure of the devices worldwide. The scenario where worldwide connectivity is basic need, MAS is supposed to be in the first row of such systems that can join worldwide connectivity and can be distributed among enough units.  Figure 4 shows MAS infrastructure.

### 2.1. MAS Infrastructure

In development of every system, there is an important role of infrastructure, the same spread on to MAS. In general, basic element of MAS is to be considered as actuator, sensor, communication network, and controller. An important thing is that MAS can be a close-loop or open-loop system. In way of IoT, the global network has access through open loop such as Big Data and cloud computing which are addition in infrastructure of MAS. Hence, MAS may contain a huge number of heterogeneous devices containing actuators, sensors, etc. In addition, it also has roughly limitations on MAS, communication technologies, for instance, and requirement of safety protocols. We can say that such heterogeneity is the main challenge in MAS, so different kinds of devices gain care from a system. To see challenges in big and small systems, among challenges, for example, unified integrity, mobility also affects the entire system. It represents that transferring of devices may cause various problems and need to be taken in regular working of the system. Infrastructure is complicated span because it consists of both hardware and software. MAS architecture is complicated summarizing cyber and physical space. MAS architecture has been studied in [20], contains five-layer MAS infrastructure. In [14], it has been studied four-level MAS architecture. If MAS needs to be in connection with worldwide/global networks, such as Internet, TCP/IP can be the best candidate in which two last layers, physical and data, accordingly are shown by the single level. Transport, network layers, and application form other three layers part. Explained categorization of physical threats in fault estimation, detection, and tolerant of MASs has been studied in Table 2.

### 2.2. MAS Virtualization

In MAS, the basic purpose of virtualization is to abstract or hide complicated detail such as technical detail from above laying layers and to permit stretchy sharing resource, so that resources or working given as facilitation. So, MAS joints physical and cyber space and contains throughout process from signal to complexity of applications. There are some virtualization techniques such as network virtualization which is divided into subtypes such as router virtualization, network interface card virtualization (NICV), and link virtualization, application virtualization, and device virtualization.

### 2.3. Consensus Information

Suppose a decision-making agent network with ${\dot{x}}_{i}=u_{i}$ attentive in accessing via local communication with nearby on *G* = (*V*, *E*). By accessing consensus, it seems asymptotically joining to single-dimensional contract space characterized by

(1) $$\begin{array}{c} x_{1}=x_{2}=,\ldots =x_{n}. \end{array}$$

Such contract can be represented as *x* = *α*1 in which 1 = (1,…, 1^*T*^) and *α* ∈ *ℝ* is joined decision of all agents in the group. Suppose *A* = [*a*_ij_] is adjacency matrix of *G* graph. Agent *i* set in nearby is *N*_*i*_ and represented as

(2) $$\begin{array}{c} N_{i}=\left\{j\in V:a_{\text{ij}}\neq 0\right\};V=\left\{1,\ldots ,n\right\}, \end{array}$$

where *i* agent communicate with *j* agents which is neighbor of *i*. All set nodes and nearby agents represent *E* edge set of graph as *E* = {(*i*, *j*) ∈ *V* × *V* : *a*_ij_ ≠ 0}. *G*(*t*) = (*V*, *E*(*t*)), and a dynamic graph is that where edges set *E*(*t*) and *A*(*t*) adjacency matrix varying time. *N*_*i*_(*t*) nearby set in the dynamic graph of each agent is well. Such dynamic graphs are helping for explaining the mobile sensor technology network and flocks. The linear system is

(3) $$\begin{array}{c} {\dot{x}}_{i}\left(t\right)=\sum_{j\in N_{i}}a_{\text{ij}}\left(x_{j}\left(t\right)-x_{i}\left(t\right)\right). \end{array}$$

It is a distributed consensus algorithm, and it follows that sum of state of all node is an invariant quantity. When applying this condition at time *t* = 0 and *t* = *∞*, we get

(4) $$\begin{array}{c} \alpha =\frac{1}{n}\sum_{i}x_{i}\left(0\right), \end{array}$$

while in another way, if there is asymptotically access of consensus, then mandatory cooperative result is equal to initial state average of all nodes. With such variance characterization, the consensus algorithm is known as average consensus algorithm and has several applications in distributed computing on networks such as sensor fusion in the sensor network.

In compact form, system dynamic can be represented as

(5) $$\begin{array}{c} \dot{x}=-Lx, \end{array}$$

where *L* = graph Laplacian *G* and it can be defines as

(6) $$\begin{array}{c} L=D-A, \end{array}$$

where *D* = diag(*d*_1_,…, *d*_*n*_) is degree matrix of *G* with zero off-diagonal elements.

*L* has right eigenvector of 1 linked with zero eigenvalue because of the identity *L*_1_ = 0.

## 3. Attack Modeling and Methodology

### 3.1. Approach of Modeling DoS Attack

We will study two important techniques for DoS attacks modeling in MASs: one is queueing technique and also another is stochastic technique.

#### 3.1.1. Queueing Technique

Like computers, firewalls and routers are considered networking devices, providing poor performance in supervision of DoS attacks, though trade with maximum rate because of memory resource constrains, interrupt processing, and input output processing and central processing units. However, packet loss and delay jitter are vastly affected under attack which can cause disturbance to the control system performance, e.g., mean squared error, rise and settling, and overshoot of percentage. The transmission of packet in the network control system under DoS attack is approached to smear simple techniques which are based on several input queues [35–37]. Two techniques are discussed as follows:

1. DoS attack is launched by attackers to an endpoint from the system or PC to surrounding area nearby to endpoint. In this way, a huge number of packets are lost.
2. DoS attack can be launched by attackers with the use of remote system to initial edged routers foremost to leisurely down network connection between controller and a remote plant.

DoS is considered as singularity that can save control signal from required time preserved. It is done by single host. It shows that control and measurement channel may be provoked individually. Hence, it can be supposed that in the process of DoS attack, it seems complicated to receive or send data. Suppose {*m*_*j*_}._*j*∈*X*_0__ in which *m*_0_ > 0, which is DoS 0/1 transition sequence; here, 0 is for “off” and 1 is for “on” situation, e.g., that time when DoS variation a transition from 0 to 1 and that time there is possibility of interruption of communication, so

(7) $$\begin{array}{c} M_{j}≜\left\{m_{j}\right\}W\left[m_{j},m_{j}+\rho_{j}\right]. \end{array}$$

*M*_*j*_ is representing time interval of *j*th DoS attack. That length may be *ρ*_*j*_ ∈ R_≥0_, this is the time when there is no communication, consider *ρ*_*n*_ = 0, and here, *j*th DoS attack is shown as individual pulse at *m*_*j*_ time. An input is generated by an actuator which is based on fresh received controller data through DoS attack. Given *ρ*, *φ* ∈ R_≥0_with *φ* ≥ *ρ*, suppose that

(8) $$\begin{array}{c} ∴\left(\rho ,\varphi \right)≜\underset{j\in X_{0}}{∐}M_{j}\prod \left[\rho ,\varphi \right],\\ \frac{{}^{\circ}\rho \varphi ≜\rho \varphi }{∴\rho \varphi }. \end{array}$$

It represents that at each interval [*ρ*, *φ*], ∴(*ρ*, *φ*), °(*ρ*, *φ*) and [*ρ*, *φ*] are representing to time instants set, when communication is permitted and stopped, respectively. Applying control signal to all *ρ* ∈ R_≥0_, it can be written as

(9) $$\begin{array}{c} w\left(\rho \right)=\text{PQ}\left(\rho_{P_{\left(\rho \right)}}\right),\\ w\left(\rho \right)=\text{PQ}\left(\rho_{P_{\left(\rho \right)}}\right). \end{array}$$

It shows that for all *ρ*_*j*_ ∈ R_≥0_.*P*(*ρ*) modern fruitful control approach, the same to proposed techniques is used in [38]. In concluded form, we can say that there is disadvantage of this start and end of approach is not found and is most useful to post records.

#### 3.1.2. Stochastic Technique

Generally, cyberattacks are performed when the system is weak to detect the threat and results in security defilement. In practice, such attacks are introduced by the series of actions to compare security services such as confidentiality, integrity, and availability of MAS applications such as telecommunication, military, banking, smart power grids, and transportation systems. To trace the threats and cyberattacks such as file-less malware, advance persistent threat, and zero days, CPS has become focus of interest. Other than this, a number of techniques were introduced to predict cyberattacks against MAS, in which several techniques have been developed using a stochastic approach such as Hawkes process model, Markov chain model, negative binomial distribution model, and Poisson model. In the fast-growing trend of CPS attacks, attacks are considered to be launched externally against MASs within a given amount of time and using stochastic distribution models such as Bernoulli model [38, 39] and Markov model [40]; from the LTI system, the Bernoulli model can be seen:

(10) $$\begin{array}{c} \left\{\begin{array}{c} Y\left(P+1\right)=B_{Y}\left(P\right)+\mu \left(P\right)A_{W}\left(P\right)+u\left(P\right),\\ F\left(P\right)=\gamma \left(P\right)H_{Q}\left(P\right)+h\left(p\right). \end{array}\right. \end{array}$$

For the Markov model, we supposed the following:

(11) $$\begin{array}{c} \left\{\begin{array}{c} Y\left(P+1\right)=B_{Q}\left(P\right)+\mu \left(\delta \left(P+1\right)\right)A_{W}\left(P\right)+u\left(P\right),\\ F\left(P\right)=H_{Q}\left(P\right)+h\left(P\right). \end{array}\right. \end{array}$$

From (6), *h*(*p*) = measurement noise and *u*(*P*) = process noise.

These measurement noise and process noise are commonly known as independent and identically distributed. Here, with 0 mean Gaussian random vector and covariance *Q*, *μ*(*P*), *γ*(*P*) are independent identically distributed. Bernoulli is relevant to existence of DoS attack on measurement and process noises [41].

Now, see (7).

*μ*(*ð*(*P* + 1)) ∈ {0,1} is known as Markov controlled DoS attack sequence which stops transmitting of control signal packets to actuator in which *ð*(*P*) is similar to interior state of attacker [42].

## 4. Fundamental Issues of Multiagent Systems

**Figure 5:**
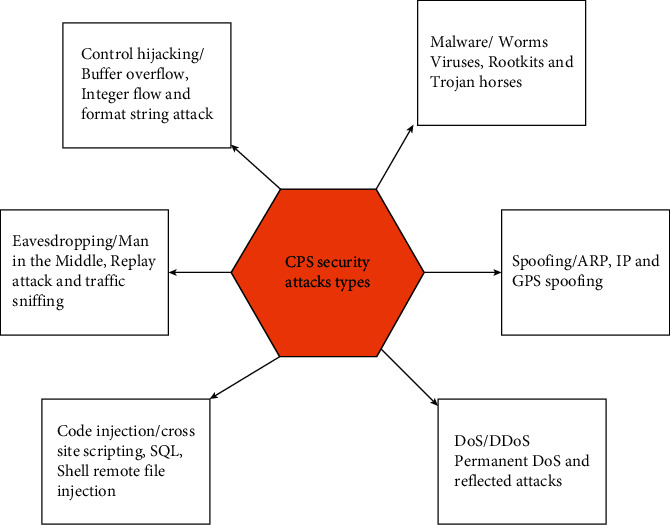
Types/subtypes of MAS security attacks.

Basic purpose of MAS classification is to explain overview and fundamental issues regarding cyberattacks. Some general examples of cyberattacks are distributed denial of service (DDoS), man-in-the-middle (MITM), deception attack, password attack, and malware attack. Cyberattack is an offensive action, while if there is possibility of occurring of attacks, then it is known as cyber threat, while cyber risk is interconnected with the word threat which estimates the probability of proportional loss which may occur. Figure 5 shows basic types and subtypes of security attacks.

**Figure 6:**
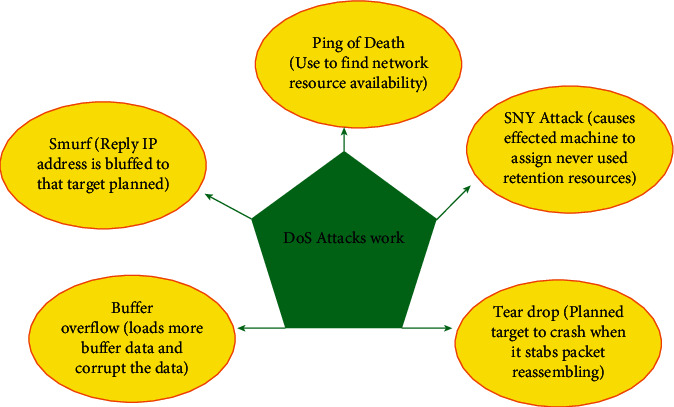
Working of DoS attacks.

Here, we will discuss few cyberattacks and fundamental issues discussed in latest research. Figure 6 shows working of MAS security attacks.

### 4.1. Deception Attack

There is hastily emerging phenomenon of use of deception technology in contemporary cyber security as a feasible means active and intelligent postbreach defense. Similar to any unruly technology it happens with fallacies. Cyber security needs to be changed from being dependent on largely detecting untrue things within a cloud of healthy activity to being focused on stopping cybercrime, which tends to tempt, phish, deceive, and trap users. Deception tactic often proves to be healthy for defense and attack. Deception technology has progressed far yonder the honeypot perception. Now a day's deception is being active in baiting and luring attackers to a deception environment. Deception, also known as malicious attacks and false data injection (FDI) attacks, is defined and studied in [9, 43–45], e.g., nominated malicious system Stuxnet which is able to be reprogrammed and running code in PLCs in SCADA system cause aberration from required conduct. In power grids, transmission system adversaries can send attack to hack remote terminal units, e.g., in substations, there are sensors [12]. For another example of such kind of attack, see study [14, 46, 47]. Deception changes cyber security by providing sole breadcrumbs and traps for industry specific environment, legacy system, IoT, and devices where low cost regularly excludes security structures. Authors in [48] considered measurement output to encounter deception attack based on Bernoulli distribution during signal transmission. To describe random property of deception attack, Bernoulli distribution has been deployed [14]. Deception attack has been used in the term of limited time boundness [14].

### 4.2. DoS Attack

Denial-of-service (DoS) attack denies or makes slow to the authentic users to access a resource web, e.g., emails and network. DoS attack is policies, and those are usually used for profession of communication capitals in order to forbid the measurement transmission and cause supreme possible worsening of performance of the system. The common DoS model has been studied in [49] in which DoS topographies are discussed with DoS duration and DoS frequency. Similarly, improvement in this idea has been studied for production with the output controller of dynamic feedback.

Complicated form of DoS is distributed denial of service (DDoS) [50, 51] which is also known as coordinated attack, where a huge number of cooperated machines work to achieve DoS attack [52]. However, because it can be easily created, so DDoS is easily available, has high impact and low cost of systems, consisting ability of fully detach an association [53]. It is represented that there is instability of power grids because of attacks and could give long delay jitter on network control system packets. The division of DoS attack in radio frequency identification is because of the reasons studied in [54, 55], e.g., desynchronization attack, system jamming, kill command attack, tag data modification, and random DoS attack as shown in Figure 5.

In conclusion of this DoS attack, aforementioned forms of DoS attacks are implemented for the classification of DoS attack in radio frequency identification systems as studied in [29, 54, 56]. Therefore, they could be present in many forms of MASs.

### 4.3. Jamming Attack

Such DoS attack refers to condition when one channel is occupied by an attacker for prevention of other node from its use which causes blocking of communication. For obtaining optimal defense mechanism for the network control system, stochastic game theory is applied [29, 57, 58]. Dynamic collaboration among attackers and sensor transmitters in the network control system was projected as the double-player stochastic game. In stochastic game, cost functions contain source cost used for conduction of attack actions, cyber-layer defense, and as possible harmed dynamic act of the network control system. Interaction effect between defender and attacker on dynamic concert of the network control system was supposed by the following cost function. Finally, a stochastic dynamic programming delinquent has been explained for gaining optimal defense mechanism.

In [59–61], security in remote state approximation of MASs has been studied. Communication between remote approximator and sensor node was taken through wireless channel that may be attacked by a jamming attacker. Best decision of process making of both attacking and communicating was discussed in case of consideration of energy constrains for both attacker and the sensor. Markov theory was used for gaining equivalent solutions, and constrained relax delinquent was designed.

For maximization of linear quadratic Gaussian used optimal jamming attack, it controls cost function while supposed energy constraints studied in [59, 60, 62]. Corresponding cost and optimal jamming agenda were consequent after studying the usage of cost function under a free attack agenda. The fresh analytically model was studied in the influence of attack jamming on broadcasting. A jamming attack for optimal energy efficient by wireless channel under jammer attacker energy constraints is studied in [63].

These attacks forced by power constrained pulse width-modulated jammers are supposed to be moderately recognized, which is jammer period and unchanging inferior destined and jammers asleep periods are identified. Controller synthesis problem that is an event based for network control systems and strong event triggered communication scheme was studied in [27]. In conclusion, piecewise Lyapunov function is applied to guarantee exponential stability of the system.

### 4.4. Man-in-the-Middle (MITM) Attack

Generally name “man in the middle” is derived from a game scenario known as basketball, where two players aim to throw a ball to each other, and one of them tries to clutch it [64]. MITM is well-known computer security attack, which gives a great challenge to security professional. Actually, it hits real data flowing between confidentiality, endpoint, and integrity of the data. For analyzing and categorizing the MITM scope, researchers should read [65] survey.

Basically, MITM takes benefit of authentication protocol weakness used by communication networks. Usually, the third party is responsible for authentication that issues certificates because the system of certificate production becomes another way of solid weakness. MITM attacks permits unauthorized parties to snoop data by backdoor.

#### 4.4.1. Example of MITM Attack

About in 2015 [66], it was known that Lenovo machines are available with already installed adware known as superfish which injects adds on browser such as Internet Explorer and Google Chrome. Superfish installs a compiled certificate into Windows certificate database. It leaves all secure stock layer (SSL) certificates given by Hypertext transfer protocol secure (HTTPS) that are linked with personal certificates. Such situation may provide an access to hackers to receive sensitive information such as bank account information, transactions, or user lifestyle..

Internet of Things (IoT) is progressing from smart home to smart cities and making our lives dependent. With the passage of time, billions of M2M will be interconnect with each other, and the big problem is to manage such a big problem for network administrator. Intensive-security methods, classical computing method as antivirus, and encryption are not directly installed software. With network infrastructure, it is compulsory to make IoT devices more secure.

Opposite to the traditional security network, software define network (SDN) [63] gives several new features, as centralized control and network programmer, which skilled the owner to manage network automatic in a dynamic and flexible way. We can see that IoT future [67] is SDN dependent. Open floe channels security issues of IoT, e.g., MITM, are studied in [68].

**Figure 7:**
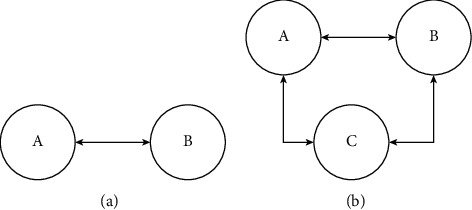
Victim with and without attack.

Distributed methodology for agent network permission targeted for execution of the distributed algorithm to control MITM attack is studied in [69, 70] which intends steering algorithm result towards erratic values of risky configurations. An example of MITM attack is shown in Figure 7.

Figure 7(a) shows victims without attackers, and Figure 7(b) shows victims with attacker, contenting message between A and B without notice.

#### 4.4.2. Key Points

There are following key points of MITM:

1. MITM permits hackers to intercept confidential data.
2. MITM is session hijacking type.
3. MITM permits hackers to insert malicious data and sites in form misty from genuine data.
4. MITM exploits real-time nature of data transfer and communication to go hidden.
5. MITM contains hackers inserting themselves as proxies or relays in an ongoing data transfer or appropriate conversation.

Researchers can study the following literature for their research interest of MITM attack [71–73].

#### 4.4.3. EECDH Prevention Technique

Enhanced Elliptic Curve Differ-Hellman (EECDH) prevention technique for MITM attack is well studied in current research [45] which improves the security level. Keep secure MITM attack where communication carrier clears themselves before cooperating their keys, to use Differ-Hellman key exchange for communal verification, so that during cloud sharing, data privacy sustained.

### 4.5. Replay Attack

**Figure 8:**
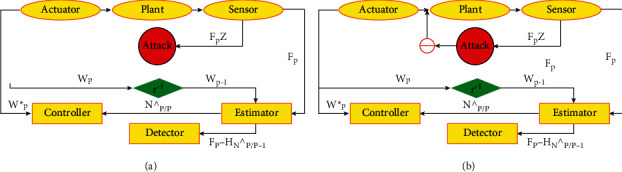
First and second phase of replay attack.

Such kind of deception attack happened when adversary succeed in recording some of the transmission data, e.g., in MAS, sensing data are injected [74, 75]. This form of attack is supposed to happen in two ways, e.g., in 1^st^ way of Figure 7 recording data of attacker from system and injecting the same data in the system, and another way is attack could be outside carrying that subject to physical systems represented in Figure 8 [76]. Likewise, an attacker formed communication connection in between two last points to enclosure observed messages in various areas in globe generally present in WSN [77]. In designing, such attack could be assumed as changeable delays with unidentified data on variable rates and upper bounds. Applying time-delay system concept jointly by optimization methods acceptable max upper bound can be premeditated [3].

There is no requirement of system information in such form of attack, containing information on designed estimator and controllers; for detection, this activity makes it complicated. Adopting counters and time-stamp in the transmitted data is solution opposite to such attack. Two phases, first and second of replay attack, has been shown in 8.

There is not enough research that studies controlling of MASs subject to replay attacks, e.g., recording vista control variation direct to replay attack is discussed in [78], which gives an explicit and simple connection in between computing, attacking horizons, and infinite-horizon cost. Then, asymptotic exponential stability of the system is ensured by availing enough condition set; see one more example study [74, 79]. Feasibility terms of replay attack and countermeasure suggestions those enhance the possibility of detection by supposing control performance are discussed in [80]. And integrity attack on the control system is countermeasured and analyzed proficient of showing these attacks were not assumed. For further example of such attack, we can see [79, 81, 82].

## 5. Agents Communication, Faults, and Simulation Environment for Agent-Based MAS Network

In this section, we will study MAS agent communication, faults, and simulation environment.

### 5.1. MAS Agent Communication

We know that MAS contains self-directed agent group which works in cooperation with each other through other communication medium to gain considered goals, and find number of usage in different areas such as physics, biology, mathematics, social science, and computer engineering.

**Figure 9:**
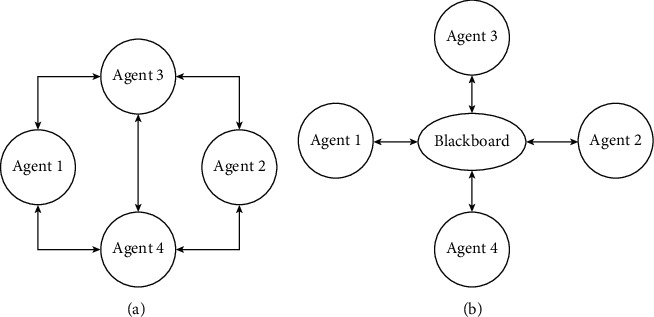
An overview on communication approaches in MAS.

Since 1962 [83], agent's communication has been studied. Mainly used communication approaches are message passing, speech act, and blackboard. In message passing, agents directly message each other as shown in Figure 9(a). There is use of broadcast or point to point agent communication to communicate with other agents. In the broadcast communication, one agent sends message to all nearby agents while in former agent one can directly talk to another agent in case of other agent address information.

In speech act, researchers in [84] studied that some sentences or utterance verbs are act as speech acts that vary physical environment such as in general environment if general person uses sentence that “I now make you man and wife” such kind of sentence have impact on physical environment by introducing new condition and rule. Agent action can be as a speaker, which produces utterance to vary listener belief [84, 85].

In blackboard communication, agents share data with each other in collaboration by use of central repository known as blackboard, Figure 9(b). In this, data of each agent are stored in blackboard which are accessible and readable by other agents. Blackboard uses control information for controlling agent's access. It is important for message semantics that need to confirm communicating agents with each other who have the same understanding of exchanged data. Sometimes in heterogeneous agents, it can be a challenging task.

### 5.2. MAS Agent Communication Faults

Basically, for MAS, two kinds of faults are considered known as network fault and agent fault. Network fault occurs in the communication network and may disturb communication performance while agent fault that can occur in internal components of actuators, agents, and sensors. There are three types of classification of agent's faults, sensors faults, agents fault, and actuator fault which may affect agent dynamic.

### 5.3. Simulation Environment for Agent-Based MAS Network

Here, we are studying various evaluations and modeling methods used for metrics performance that differ depends upon task and MAS application of the considered agent-based system in comparison with state of the art. There are three basic evaluation methods, MATLAB, Java agent development framework, and GAMA.

Using MATLAB, we study MAS performance, especially with mathematical complex environment. In addition, it is adjustable to Java agent development framework for more work on MAS performance.

Java agent development framework is mostly used among simulators in MAS. Its admiration stalks from following properties. It benefits from third-party libraries and also is Java based. It is also written on foundation of intelligent physical agent's standard. For designing MAS, it has graphical interface. It supports simulation distributed systems, is open source, and can link to Matlab, and also it skins complexity of MAS.

Third, GAMA is simulation and modeling platform for agent-based system development. There are some advantages of GAMA such as it supports widely level MAS that contains a huge number of agents, it is useful for simulation purpose of any kind of MAS application, and it supports intuitive agent-based language such as GAML.

Studied simulation methods are specifically for MAS, while because of large-level usage, specific evaluation methods are used for system analyzing in particular application and can be deployed for agent-based system simulation performance to see issues in that application.

This is an important method which is usually used in the deception attack in sensor networks which contains the hypothesis test with predefined probabilities of binary hypothesis [86, 87]. Cooperative spectrum sensing performance limit is evaluated subject to Byzantine attacks; however, false data affect the fusion center, because of that output of wrong sensor increased [88, 89]. On binary hypothesis, a similar ratio detector is considered to manage with already determined fixed error for security of smart grid in sensor network data [20, 87, 90], energy frame work that is deep learning based, and block chain based is well studied in [20, 89]. A detector based on progressed similar has been studied in [91–93] and supposed for unobservable and observable circumstances in the SCADA system. An example of such methods has been applied in [94]. Important hypothesis applied is that all node transmitted packets are arbitrary; by this way, probability of next packet will not affect in verdict a packet to be nasty. In conclusion, there can be several forms of attacks those can affect single or several packets. For calculation of trust values, it is to be considered that node is transferring “*X*” packets; here, *j* packets are supposed to be normal. Observation of *x*(*X*) = *j* distribution is studied by given binomial distribution:

(12) $$\begin{array}{c} K\left(x\left(X\right)=j|K\right)=\left(\begin{array}{c} X\\ j \end{array}\right)K^{j}{\left(1-k\right)}^{X-j}. \end{array}$$

Here, *K* shows that *i*^th^ packet probability is normal k(K) = no. of normal packets this model intends to guess the probability of *K*(*W*_*X*+1_ = 1|*x*(*X*) = *j*) and result out either the *X* + 1 packet is in normal form. With the use of Bayesian theorem, the following probability distribution is calculated:

(13) $$\begin{array}{c} \left(K\right)\left(W_{X+1}=\frac{1}{x\left(X\right)},=j\right)\\ =K\left(W_{X+1}=1x\left(X\right)=j\right)K\left(x\left(N\right)\right.=j. \end{array}$$

Here, we can apply marginal probability distribution:

(14) $$\begin{array}{c} k\left(x\left(X\right)=j\right)=\int_{0}^{1}k\left(x\left(X\right)=p|k\right)g\left(k\right).vk,\\ K\left(W_{X+1}=1,x\left(X\right)=j\right)=\int_{0}^{1}K\left(x\left(X\right)=j|k\right)g\left(k\right)k.vk. \end{array}$$

There are no data for *k*; now, it is considered that it can be found by uniform prior distribution *g*(*k*) = 1; here, *k* ∈ [0,1]. Hence, we can rewrite above equations (12)–(14) as

(15) $$\begin{array}{c} K\left(W_{X+1}=1,x\left(X\right)=j\right)=\frac{\int_{0}^{1}K\left(x\left(X\right)=j|k\right)g\left(k\right)k.vk}{\int_{0}^{1}k\left(x\left(X\right)=p|k\right)g\left(k\right).vk},\\ =\frac{p+1}{X+2}. \end{array}$$

In resultant from equation (15), both of normal packets number *j* and *X* which is whole packets can be found in WSN, after the collection of traffic information. By applying suitable threshold, we can find malicious node. Some numerical results of such malicious nodes has been studied in [92, 95]. With the use of this model, we can find malicious node.

### 5.4. Weighted Least Square (WLS) Approach

It is an effectual consistent attack detection method for dimension data. Mostly, it is applied in power systems and smart grids [96–99]. By comparison of predefined threshold and constructed measurement residual, we carried out a bad resultant.

Suppose

(16) $$\begin{array}{c} r=MY+c. \end{array}$$

Here, *M* = [*m*_ab_]_*i∗j*_ is known as measurement Jacobian matrix with full column rank, when *j* > *x*, *m* and *r* are considered states vector and measurement, respectively, and *c* is system noise effecting. Estimated delinquent is used to solve the *m*^∗^ of variable *m*, which is better for measurement of meter *r* w.r.t equation (10). Estimated measurement *r*^∗^ and observed measurement have a difference that is defined as *z* = *r* − *r*^*∗*^ = *r* − *MY*^*∗*^. WLS problem is to find an estimate *m*^∗^ which slow the index performance *D*(*Y*^*∗*^), which can be found by the given formula:

(17) $$\begin{array}{c} \min_{y*}D\left(Y^{*}\right)≔{\left(r-MY^{*}\right)}^{Z}U\left(r-MY^{*}\right). \end{array}$$

In this weight matrix, *U*≔∑−1.

To simulate 1^st^ order optimal situation, *D*(*Y*^*∗*^) is studied in [43]:

(18) $$\begin{array}{c} y^{*}\left(M^{Z}UM≔Cr\right), \end{array}$$

where “*C*” is pseudoinverse of *M* and *CM* = 1.

### 5.5. *Q*^**2**^ Detector Kalman Filters

Here, we used characteristics of Kalman filter residual instead of WLS, to make it feasible for good or bad data:

(19) $$\begin{array}{c} Q_{p+1}=B_{Q_{p}}+A_{b_{p}}+u_{p},\\ F_{p}=H_{Q_{p}}+h_{p}. \end{array}$$

In this, *b*_*p*_ ∈ *R*^*k*^, *Q*_*p*_ ∈ *R*^*k*^ and *F*_*p*_ ∈ *R*^*j*^ are the control input, state variable, and system measurements, respectively; *h*_*p*_ ≈ ∇(0, *R*)and *u*_*p*_ ∈ *R*^*k*^ are measurement noise and process noise, respectively. We can calculate *Q*_*p*/*p*_^*∗*^ with the use of given Kalman filter:

(20) $$\begin{array}{c} N_{0|-1}^{*}=N_{0}^{*},\\ K_{0|-1}=\varepsilon ,\\ Q_{p+1}^{*}=B_{Q_{p}}+A_{b_{p}},\\ K_{p+1|p}=BK_{p}B^{Z}+S,\\ P_{p}=K_{p|p-1}{\left.H^{Z}+R\right)}^{-1},\\ Q_{p}^{*}=K_{p|p-1}+P_{p}\left(F_{p}-HQ_{p|p-1}^{*}\right),\\ K_{p}=K_{p|p-1}-P_{p}HK_{p|p-1}. \end{array}$$

It has been known that Kalman filter exists in *F*_*i*_ − *HQ*_*i*|*i*−1_^*∗*^ for equation (19), with LQG controller, and Kalman filter is Gaussian independent identically distributed [22]. Suppose

(21) $$\begin{array}{c} e_{p}≔\sum_{i=p-\text{T}+1}^{p}{\left(F_{i}-{\text{CQ}}_{i|i-1}^{*}\right)}^{Z}\sigma^{-1}\left(F_{i-}{\text{CQ}}_{i|i-1}^{*}\right). \end{array}$$

Here, *e*_*p*_ = has an *Q*^2^ distribution among *jT* independent degree in normal operation which shows the lower probability of greater *e*_*p*_. *T* is window size. Here,

(22) $$\begin{array}{c} e_{p}{\text{⁣}}_{{>}_{M_{1}}}^{{<}^{M_{0}}}\rho . \end{array}$$

where *M*_0_ represents null hypothesis, *M*_1_ represents under attack hypothesis, *ρ* represents threshold discussed in [100, 101] used for the SCADA system, and the same results have been shown in [102]. *Q*^2^ detector with cosine has been explained in [95, 103, 104] for the detection of false data injection attacks which affects smart grid. An algorithm is discussed for the detection of deception attack in an application which could be remote state application, smart sensors used for data receiving [105]. Second application of such kind has been studied for the detection of bias injection attacks for stochastic rectilinear dynamical scheme [12, 106–109]. Multiclass support vector machine was discussed for building an intrusion detection model.

### 5.6. Quasi Fault Detection and Isolation Techniques (FDI)

FDI is famous and widely used in the networked control system. It identifies the fault presence, location, and fault type because it contains the monitoring system. This technique is helpful in detection of exterior attack in MASs. Attacks were supposed as unidentified inputs which effects both of states and measurements [110–113]. Using graph theory, undetectable attacks were characterized and also with the use of distributed and centralized monitor have been planned for the detection of distinguishing attacks. Based on geometric approach, a fault detection method has been implemented for detection of cyberattacks and fault in power systems or networks [114, 115]. The common system was applied for the detection of deception attacks and sensor actuator [44, 114]. Likewise, a model diagnosis system and free-fall detection were studied for designing cyberattack detector for the distribution system of water [26]. For detection and differentiation of both cyberattacks and fault, an intelligent generalized predictive controller was intended [25, 116]. Cyberattack can be targeted by recognized weakness in the system, which is weak point of FDI, different from losers commonly arbitrary or random. For designing the required robust system, this technique needs cautious investigation in order. In conclusion, it is important because of highly study, which lot of research has been done on for detection of cyber security in power systems, e.g., [117–120].

#### 5.6.1. Argument

Bayesian detection with binary hypothesis has been broadly studied and pragmatic in sensor network data fusion [23, 121,122]. In meaning of state approximation, there is need of system noise in a stochastic framework for the attack detection method, because it allows a probabilistic state approximation [64, 123]. Hence, there is need to smear mean and variance for state disruption distribution shown as freely variables. Providing of confident state approximation is important in many present applications, e.g., system guidance and navigation, target tracking, and attack [64, 123]. In short, modeling the state distributions in some sets supposed to be unidentified, but limited noises are more suitable. Therefore, *ℵ*^2^ is mostly useful based on holding discrepancies in approximation conduct which forecast by a model. Resultant unidentified but limited sounds are suboptimal, and attack detection feasibility is reduced.

## 6. Cooperative Attack Methodologies

Comparing to other kinds of attacks, e.g., DoS attack and replay attack, we got no much more interaction of researchers. This is because of phenomenon that fixed on controlling of MASs. Both defending and detection against DoS attacks in a state approximation delinquent were studied in [124, 125]. Data of sensor are transferred to the estimator by a packet, falling communication network. Already defined Kalman filtering [126, 127] for approximation of state for the untrustworthy communication system is pragmatic in this network. First of all, hypothesis testing detection problem is articulated, while supposing already known knowledge of network statics. Secondly, there were considered two preventing policies with the use of secure coding packet slant for recompensing the absence data, and another is improved on transmission power up gradation to control the blocking upshot of attack.

In [126, 127], game theory approach has been studied, e.g., collaboration between attacker and sensor is designed as zero sum stochastic game, which finger to DoS attack in remote state approximation. Presence of Nash equilibrium was primarily studied for such kind of game, and later on the best policies were planned for fixing sensor transmission power. For calculation of asymptotic performance of remote approximator, planned form game is applied in [128, 129].

For nonlinear chaotic systems, sliding mode control with actuator fault and decentralized sliding mode for heterogeneous MASs problem of fault tolerant control considering both DoS and network fault is discussed in [25, 131]. It is to be supposed that network fault contains of deterioration, signal attenuation, and perturbations of couplings those are in nonlinear form. Reimbursement of perturbed couplings and faulted were gained to apply a strategy which is known as slide mode planned strategy; by way of unidentified constraints of approximation, then the mathematical analysis method and Lyapunov stability theory were applied to assurance the asymptotic management of the nonlinear confused system. We see another example of approximation of delinquent in MASs exposed to DoS attack studied in [7, 130–132].

### 6.1. Secure Control Approaches for DoS Attack

**Figure 10:**
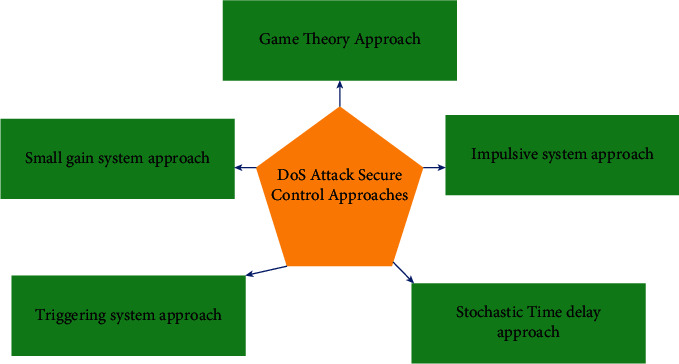
Secure control approaches of DoS attack.

For controlling MASs exposed to DoS attack, many researchers worked; those are shown in Figure 10.

#### 6.1.1. Stochastic Time-Delay System Approach

Here, DoS is designed as stochastic process with signal delay. Deception and DoS attacks are supposed to be freely stirring and designed as Bernoulli distributed white sequences in [12, 131]. Suppose the discrete time stochastic system with measurements and noise effecting the system, as

(23) $$\begin{array}{c} \left\{\begin{array}{c} Q_{P+1}=\left(B_{0}+\prod_{i=1}^{z}\left.\tau_{a,p}B_{a}\right)Q_{P}+{\text{BW}}_{P}\right),\\ F_{P}=\left(H_{0}+\prod_{a=1}^{r}\tau_{i,P}H_{a}\right)Q_{P}. \end{array}\right. \end{array}$$

Here, *F*_*P*_ ∈ *R*^*x*_*f*_^ is measurement of sensor and *Q*_*P*+1_ ∈ *R*^*x*_*m*_^ is state vector, and *W*_*P*_ ∈ *R*^*x*_*w*_^ is input of controller. *B*_*a*_(*a* = 0,1,…, *z*), *A* and *H*_*a*_(*a* = 0,1,2,…, *l*) are with appropriate dimension and constant matrices. *τ*_*a*,*p*_ ∈ *R*(*a* = 1,2,…, *z*) and *τ*_*a*,*p*_^*∗*^ ∈ *R*(*a* = 0,1,2,…, *l*) are multiplicative noises with unity variance and zero means, and jointly uncorrelated with *P* and *a*, *z* and *l* those are positive integer, A rank is considered to be *x*_*w*_, and to study this kind of problem, we see the given model of attack:

(24) $$\begin{array}{c} F_{P_{r}}^{\varphi }=\left(\mu_{p_{r}}^{≅\varphi }+\partial_{P_{r}}^{\varphi }h_{P_{r}}^{\varphi }\right)+\left(1-\mu_{P_{r}}^{\varphi }\right)F_{P_{r-1}}^{\varphi }, \end{array}$$

where *F*_*P*_*r*__^*φ*^ = data received by controller and *h*_*P*_*r*__^*φ*^ ∈ *R*^*x*_*f*_^ = stands for attackers injected signals as in (23):

(25) $$\begin{array}{c} h_{P_{r}}^{\varphi }=-F_{P_{r}}^{≅\varphi }+{ð}_{p_{r}}^{\varphi }, \end{array}$$

where *ð*_*p*_*r*__^*φ*^ is freely fixed signal which satisfies

(26) $$\begin{array}{c} \left\|\delta_{P_{r}}^{\varphi }\right\|\leq {\text{epsilon}}_{2},\\ \text{Prob}\left\{\mu_{P_{r}}=0\right\}=1-\hat{\mu },\\ \text{Prob}\left\{\mu_{P_{r}}=1\right\}=\hat{\mu },\\ \text{Prob}\left\{\partial_{P_{r}}=0\right\}=1-\hat{\partial },\\ \text{Prob}\left\{\partial_{P_{r}}=1\right\}=\hat{\partial }. \end{array}$$

*μ*_*P*_*r*__ and ∂_*P*_*r*__ = white sequences Bernoulli distributed with 0 and 1 value. Probability is given in (26). In (26),∂_._ ∈ [(0,1)]and ∂ ∈ (0,1) are identified constants. Some enough states are gained to confirm the security needs of the system in which we gained some enough conditions.

#### 6.1.2. Impulsive System Approach Hybrid Model

Here, we showed a system that is beneath DoS attack showed by the impulsive system. Resilient control techniques and resources aware are designed with malicious DoS attack studied in [133–135]. Specifically, an event-based control scheme which is output that is output based was pragmatic to control to get the communication strategy and control in lass of nonlinear feedback systems which is provoked by exogenous troubles.

Suppose

(27) $$\begin{array}{c} p:\left\{\begin{array}{c} {\hat{Q}}_{K}=g_{K}\left(Q_{K},W,U\right),\\ F=f_{r}\left(Q_{K}\right), \end{array}\right. \end{array}$$

where *p* = plant. Here, *U* ∈ *R*^*x*_*u*_^ = uproar input, *F*^*∗*^ ∈ *R*^*x*_*f*_^ = state vector, *W* ∈ *R*^*x*_*W*_^ = control input, and*F* ∈ *R*^*x*_*f*_^ output of plant:

(28) $$\begin{array}{c} £:\left\{\begin{array}{c} {\hat{Q}}_{q}=g_{q}\left(Q_{q},\hat{F}\right),\\ W=f_{K}\left(Q_{q},\hat{F}\right). \end{array}\right. \end{array}$$

In this, *Y*_*c*_ ∈ *R*^*x*_*q*_^ = controller state, *F*^*∗*^ ∈ *R*^*x*_*f*_^ = fresh gained measured output, and *W* ∈ *R*^*x*_*w*_^  = controller output; resultant output is *r* = *e*(*Q*) in which *r* ∈ *R*^*x*_*r*_^ and *Q* = (*Q*_*k*_, *Q*_*q*_). Here, attack is DoS and interval of attack is represented by {*M*_*x*_}_*x*∈*X*_ ∈ *I*_Dos_; at this time period, there is no communication between controller and sensor since attack. DoS attack collection time is given as

(29) $$\begin{array}{c} \emptyset ≜\sum_{x\in X}M_{x}. \end{array}$$

To apply framework, hybrid model *F*^*∗*^ updating can be

(30) $$\begin{array}{c} \hat{F*}=\left\{\begin{array}{c} F,\text{ When }\rho_{b}∄\emptyset ,\\ \hat{F,}\text{ When }\rho_{b}\exists \emptyset . \end{array}\right. \end{array}$$

Transmission error *c*≜*F*^*∗*^ − *f* can be

(31) $$\begin{array}{c} c^{+}=\left\{\begin{array}{c} F,\text{ When }\rho_{b}∄\emptyset ,\\ \hat{F,}\text{ When }\rho_{b}\exists \emptyset . \end{array}\right.\\ \rho_{\text{miet}}=\left\{\begin{array}{c} \frac{1}{L_{r}}\left(\frac{r\left(1-\gamma \right)}{2\left(0/0\right)\left(\left(x/l\right)-1\right)+1+0}\right),\gamma >L\text{,}\\ \frac{1}{L}\frac{1-\gamma }{1+\gamma },\gamma =L\text{,}\\ \frac{1}{L_{r}}\arctan\;h\left(\frac{z\left(1-\gamma \right)}{2\left(\gamma /\gamma +1\right)\left(\left(ℵ/L\right)+1+\gamma \right)}\right),\gamma <L. \end{array}\right. \end{array}$$

Equation (31) represents maximum allowed transmission interval limited *ρ*_miet_ that is characterized, where *z* = |(*γ*/*L*)^2^ − 1|, *L* ≥ 0 is fixed, *γ* ∈ (0,1) shows present information in local area at a mechanism known as event triggered mechanism, and we obtained *γ* as

(32) $$\begin{array}{c} \langle \nabla h\left(Q\right),g\left(g,c,u\right)\leq -\sigma \left(\left\|Q\right\|\right)-\sigma \left(\left\|F\right\|\right)-M^{2}\left(Q,W\right)-\epsilon_{1}\left(U\left(c\right)\right)+\sigma^{2}U^{2}\left(c\right)+\vartheta^{2}\left\|U^{2}\right\|\rangle . \end{array}$$

This condition explanation is well studied in [135]. At the end, to suppose DoS attack in normal form, these DoS attacks are banned in form of duration and frequency.

#### 6.1.3. Small Gain Approach

Distribution system stabilization problem exposed to DoS frequency characterization, DoS attack, and preserved stability duration is studied in [135]. To preserve communication resources, a hybrid communication technique is also supposed. By using of hybrid transmission technique, zero behavior can be saved and load communication can be compact efficiently. e.g., a large-scale system contains *X* interacting subsystem is supposed with given model:

(33) $$\begin{array}{c} {ℵ}_{a}^{*}\left(\varphi \right)=B_{a}Q_{a}\left(\varphi \right)+A_{a}Q_{a}\left(\varphi \right)+\underset{b\in X_{a}}{∐}M_{\text{ab}}Q_{b}\left(\varphi \right). \end{array}$$

In this, *B*_*a*_, *A*_*a*_ and *M*_*ab*_ are with suitable breadth. *Q*_*a*_(*φ*) and *W*_*a*_(*φ*), *φ* ∈ *R*_>0_ are control and state input of the subsystem. Input control applied to “*a*” subsystem is

(34) $$\begin{array}{c} W_{a}\left(\varphi \right)=P_{a}Q_{a}\varphi_{P}^{i}+\underset{b\in X_{i}}{∐}L_{\text{ab}}Q_{b}\varphi_{p}^{b}, \end{array}$$

where *L*_ab_ = controller coupling gain.

Suppose {*m*_*x*_}*x* ∈ *X*_0_, *m*_0_ ≥ 0 representing off/on DoS transmission, e.g., DoS displays time instant transmission from 0 to 1. Hence,

(35) $$\begin{array}{c} M_{x}≜\left\{m_{x}\right\}\cup \left[m_{x},m_{x}+\rho_{x}\right]. \end{array}$$

Equation (35) shows *x*^th^ DoS time instant. *ρ*_*x*_ is length on which there is DoS attack on the network; suppose

(36) $$\begin{array}{c} ∴\left(\rho ,\varphi \right)≜\sum_{x\in X_{0}}M_{x}\sum \left(\rho ,\varphi \right). \end{array}$$

This is subclass of (*ρ*, *φ*), and also there is DoS attack on the network.

*(1) Hypothesis A*. Equation (36) is supposed to be constant in DoS frequency, in this *τ* ∈ *R*_>0_ and *ρ*_*O*_ ∈ *R*_>0_, as

(37) $$\begin{array}{c} x\left(\rho ,\varphi \right)\leq \tau +\frac{\varphi -\rho }{\rho_{O}}. \end{array}$$

*(2) Hypothesis B*. *p* ∈ *R*_≥0_ and *Z* ∈ *R*_>1_ are present as constant in DoS duration:

(38) $$\begin{array}{c} \left|∴\left(\rho ,\varphi \right)\right|\leq p+\frac{\rho -\varphi }{Z}. \end{array}$$

*(3) Hypothesis C*. When there is no DoS attack, an intersampling interval ∇ is present, e.g.,

(39) $$\begin{array}{c} \left\|c_{a}\left(\varphi \right)P\right\|\leq \in_{a}\left\|Q_{a}\left(\varphi \right)\right\|. \end{array}$$

∈_*a*_ is appropriate design constraint.

#### 6.1.4. Deduction A

Equation (33) is representing the distributed system, and equation (34) is for control input, so for this distributed system and control input communication of a plant controller on collective network with Hypothesis C, and sampling ∇ interval. For any DoS attack, the large-scale system is asymptotically constant. Hypothesis A and B with freely *τ* and *p* and *ρ*_*O*_ and *Z* are as follows:

(40) $$\begin{array}{c} \frac{1}{Z}+\frac{\nabla_{*}}{\rho_{O}}<\frac{\tau_{1}}{\tau_{1}+\tau_{2}},\\ \left\|L_{1}\left(P\right)\right\|+\left\|L_{2}\left(P\right)\right\|\leq L. \end{array}$$

And its subsets are discussed, and second deduction detail is discussed in [136].

#### 6.1.5. Triggering Strategy

Equation (33) is representing the distributed system and equation (34) is for control input, so for this distributed system and control input communication of a plant controller on collective network with Hypothesis C, and sampling ∇ interval. For any DoS attack, the large-scale system is asymptotically constant. Hypothesis A and B with freely *τ* and *p* and *ρ*_*O*_ and *Z* are as follows:

(41) $$\begin{array}{c} \frac{1}{Z}+\frac{\nabla_{*}}{\rho_{O}}<\frac{\tau_{1}}{\tau_{1}+\tau_{2}}. \end{array}$$

We supposed a plant jammer-operator, in which communication between plant and operator is effected by jammer studied in [137]. For reduction of the system communication system, an event triggered time order was assumed. Suppose *W* ∈ *R*^*j*^ and *Q* ∈ *R*^*x*^ be input and state vector, respectively. Given system is to be supposed

(42) $$\begin{array}{c} {ℵ}^{*}\left(\varphi \right)=B_{Q}\left(\varphi \right)+A_{W}\left(\varphi \right),\\ w\left(\varphi \right)=PQ\left(\varphi_{P}\right),\;\forall_{\varphi }\in \left[\varphi_{p},\varphi_{P+1}\right], \end{array}$$

where *B*, *A*, and *P*, are proper dimensions matrices, and {*φ*_*P*_}_*p*≥1_ = triggering time sequence. Now, suppose *c*(*φ*) = *Q*(*φ*_*P*_) − *Q*(*φ*), For all ∈ ⌊*φ*_*P*,_*φ*_*P*+1_⌋, system is stable, if function *W*(*Q*) = *Q*^*Z*^*K*_*P*_ connected with ⌊*E*⌋>1 is supposed the control *W*(*φ*) which at time *Z*_*P*_ updated, to see given triggering law

(43) $$\begin{array}{c} {\left|c\left(\varphi_{P}\right)\right|}^{2}=\in \frac{\left\lfloor E\right\rfloor -1}{\left\lfloor \text{KAP}\right\rfloor .^{2}}{\left|Q\left(\varphi_{K}\right)\right|}^{2},\;P\geq 1. \end{array}$$

This law time sequence is

(44) $$\begin{array}{c} \varphi_{p,x}=\left\{\varphi_{1}\text{ satisfying above equation }\varphi_{1}\in \langle \left(x-1\right)Z,\left(x-1\right)Z+Z_{0}^{1}\rangle \right\}\cup \left\{xZ\right\}. \end{array}$$

Here, equation (42) is with asymptomatically stable, and equation (44) is triggering law.

#### 6.1.6. Deduction B

See equations (42) and (44) if assumed conditions mollifies. It is studied in [137]:

(45) $$\begin{array}{c} \frac{\left(1-\in \right)Z_{1}^{0}\left(\left|E\right|\right)-1}{2}>\left|K\right|\log\left(\mu \right),\\ \mu ≔\exp\left(\left(Z-Z_{0}^{1}\right)\alpha \left(B+\text{AP}\right)\right)+\frac{\text{AP}}{\alpha \left(A+\text{AP}\right)}*\left(\left|\frac{\text{AK}}{B}+1\right|\right)\left\{1-\exp\right.\left(\left(Z-Z_{0}^{1}\left|A\right|\right)\right)*\left(1-\exp\left(\left(Z-Z_{0}^{1}\right)\alpha \left(B+\text{AP}\right)\right)\right),\\ \alpha \left(B+AP\right)<0. \end{array}$$

Control strategy for the linear and nonlinear system with the use of the triggered method subjected to DoS attacks depends on study of ISS-Lyapunov function has been described in [138–140]. Maximum %age of time loosing response data deprived of the foremost system is instability was characterized and an event-based controller for that presence of minimum inside sampling time is definite has been supposed.

### 6.2. Game Theory (GT) Approach

GT deals with planned collaboration in between several named players and decision makes [107, 140, 141]. Each player preference order in between many options is increased in an impartial purpose for player, and all players try to optimize own impartial function. It depends on the alternate of another player in any nontrivial game, and this process of optimization depends on the selection of second players [142]. For applications of game theory in the network, we can study the literature [143–145]. For getting secure control in a lot of research studies, this method was pragmatic. Disadvantage which is because of DoS attack is designed as Markov process depends on the game among defending strategies and attack [145]. Using Lyapunov theory, four theorems were derived for assurance of the stability of system. For handling computation complexity of optimal strategies for both players, a Nash Q-learning algorithm is studied [144]. Sensor data are transmitted through a large number of channels remotely, making them vulnerable to malicious attacks. There is need to select one channel to sensor in between these paths with less probability to attack with data transmitting data. It is also decided by attackers that which channel is suitable for attack, e.g., [119]. From literature review, we can find some more examples of applying such kind of approach [142, 146].

CoFence Mechanism is assumed for DoS defense attack that endorsed “domain help domain” cooperative network between the NFV-based domain network. Furthermore, there is a dynamic resource allocation characterization for game, and we establish a game model to get incentive-compatible, effective, reciprocal, and fair resource allocation method to work on Nash equilibrium [147]. In [148], the authors supposed conflict between attacker and defender and designed a game theory framework for collective security detection.

### 6.3. Secure Control Approach

For the event triggering system or discrete time system, stochastic time-delay approach can be applied subject to arbitrary DoS attack. The system is designed using Markov process and Bernoulli process with identified statically information to govern the freely present DoS attack. In an event triggered system, impulsive system approach can be applied and is powerful in network control systems as studied in [135]. To reduce the communication in between system part triggering strategy is enough, since signal sent only specific condition of triggering is despoiled, that will minimize burden of communication.

Need of throughout information of the system is one limitation in game theory. With imperfect and incomplete information, game theory application is a developing field in network privacy and security. In addition, there is need of agents for correct estimation of security game limitations. For security measures and attack prevention, observation capabilities avail required basis [80, 149].

## 7. Model-Based Attack Methodology

There are two possible forms of occurrence of deception attacks: one is that targeted attacks which defined states are effected and random attacks where arbitrary measurements are defined [48]. In view of control engineering, it is designed as stochastic process [48, 150]. We supposed the following system for best understanding of this idea:

(46) $$\begin{array}{c} \left\{\begin{array}{c} Q\left(P+1\right)=B_{Q}\left(P\right)+A_{W}\left(P\right),\\ F^{*}\left(P\right)=H_{Q}\left(P\right),\\ F\left(P\right)=F^{*}\left(P\right)+\mu \left(P\right)h\left(P\right). \end{array}\right. \end{array}$$

*W*(*P*) ∈ *R*^*x*_*w*_^, *Q*(*P*) ∈ *R*^*x*_*n*_^and *F*(*P*) ∈ *R*^*x*_*b*_^, *F*^*∗*^(*P*) ∈ *R*^*x*_*b*_^ are the control input, states, received signal, and measured output, respectively, and *μ*(*P*) is Bernoulli distributed with deception occurrence possibility with values one and zero; so,

(47) $$\begin{array}{c} \left\{\begin{array}{c} \text{Prob}\left\{\mu \left(P\right)=1\right\}=\mu^{*},\\ \text{Prob}\left\{\mu \left(P\right)=0\right\}=1-\mu^{*}. \end{array}\right. \end{array}$$

Description of deception attack is

(48) $$\begin{array}{c} h\left(P\right)=-F^{*}+\tau \left(P\right). \end{array}$$

*(1) Note*. It is considered that data transferred by attackers mean injected fault data could be subtracted into two steps according to representation of equation (48)

−*F*^*∗*^ = for cancellation of original signal, and *τ*(*P*) is supposed to be freely limited energy signal as characterized in [150]:

(49) $$\begin{array}{c} \overset{\infty }{\underset{P=0}{∐}}\tau^{Z}\left(P\right)\tau \leq \tau^{-2}. \end{array}$$

**Figure 11:**
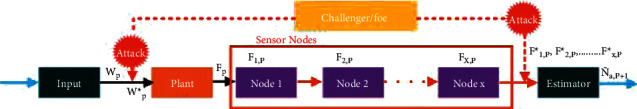
Deception attack schematic.

For the time varying class system, variance-constrained distributed problem direct to several divisions of noises, unidentified but limited turbulences, there is also study of deception over sensor network [151]. Present measurement at each node is gathered from both of neighbors and single sensors. There is insertion of deception signals into right signals of input used for controlling *W*_*P*_ and output measurements *F*_*a*,*p*_ during data transmission process shown in Figure 11. An article for designing of deception attack is studied in which nasty signals are inserted by the adversary into both measurement and control data during information communication process via network communication. Following signals effect signals.

## 8. Deception Attack Detection or Identification

It is conscious issue of deception attack estimation for prevention of any detection mechanism since attack form, the main target of affecting stability of the system. Bias injection issue hitting Kalman filter in the system containing chi-square detector is studied [152]. It proved that worst situation problem quadratically constrained can be reduced as quadratic program permits to gain criterion that is useful for selection of sensors for safe and condition on number of sensors need to keep the attack effect with encoded threshold.

Centralized security problem for stochastic system linear time-invariant with multidate-sensors fusion subject to deception attack is studied in [6]. Data transferred on each sensor by adversaries as extra signal which makes feasible boundary situations such as [25, 150]. For formulation individual rate discrete time systems, there was use of lifting technique. Using stochastic analysis techniques, enough conditions were gained for gaining already determined original system security level. For effecting uniform quantization, deception attack was supposed in distributive recursive filtering problem of stochastic system discrete time-delayed [6, 83, 153]. We supposed the following system for showing its working:

(50) $$\begin{array}{c} Q\left(P+1\right)=\left(B_{0}\left(P\right)+\overset{Z}{\underset{r=1}{∐}}\epsilon_{Z}\left(P\right)B_{r}\left(P\right)\right)Q\left(P\right)+\left(B_{0}^{\emptyset }\left(P\right)+\overset{Z}{\underset{r=1}{∐}}\in_{r}\left(P\right)B_{r}^{\emptyset }\left(P\right)\right)Q\left(P+\rho \right)+A\left(P\right)\in \left(P\right). \end{array}$$

With sensors “*x*” studied as

(51) $$\begin{array}{c} {\hat{F}}_{a}\left(P\right)=\left(H_{0}\left(P\right)+{\hat{\epsilon }}_{a}\left(P\right)H_{a}\left(P\right)\right)Q\left(P\right)+O\left(P\right)h_{a}\left(P\right),\;a=1,2,3,\ldots ,x. \end{array}$$

*Q*(*P*) state that directly cannot be observed. ${\hat{F}}_{a}\left(P\right)$ is sensor “*a*” output without quantization. ${\hat{\epsilon }}_{a}\left(P\right)$ and *h*_*a*_(*P*) are white distortions with zero means and conversance unity, collectively uncorrelated “*P*” and a, ${\hat{\epsilon }}_{a}\left(P\right)\in R$, and *ϵ*_*Z*_(*P*) ∈ *R* (*Z* = 1, 2, 3,…, *z*) are multiplicative noises with unity variance and zero means, and jointly correlated in *P*. *ρ* and *z* are positive integers. *B*_*r*_(*P*), *B*_*r*_^*o*^(*P*)and *H*_*a*_(*P*) are identified fixed matrices with well-suited dimensions. In equations (48)–(50), the same dimension effect is studied. Upper limitation for error filtering covariance has been studied in [31].

For modeling of distributed state estimator, event triggered scheme is applied to the wireless sensor network for false data injection attack [31, 154]. Each sensor estimate is checked if it attacked at all-time step before transmission of data to nearby sensor, and it may stop in case of attacked. Using event triggered scheme, an optimal estimator gain is supposed to reduce mean square estimation fault covariance, and modeled distributed estimator stability is certain with enough condition of driving. Already the discussed Bayesian method was pragmatic for both estimation and detection of states for MASs subject to turn attack signal and wrong measurements [154, 155].

Kalman filter which ensures a safe state estimation algorithm for the stochastic dynamic system was studied in [156–158]. Adversary caused freely subset of sensors is to be supposed in this problem, and an upper limit on sensors effected by attacks was designed to uphold an adequate state approximation fault. Insecure estimation situation is studied for the control system of network direct to fault data insertion attack containing a *ℵ*^2^ detector. In addition, a precise algorithm was used, and defense of rare communication channels instead of defending all is studied. Nonlinear stochastic discrete time-delay filtering problem in systems pretentious by arbitrary deception attack and arbitrary sensor saturation were studied in [159]. Suppose the system

(52) $$\begin{array}{c} Q\left(P+1\right)=B_{Q}\left(P\right)+B_{\emptyset }ℵ\left(P-\emptyset \left(P\right)\right)+\text{Ag}\left(Q\left(P\right)\right)+A_{\emptyset }g_{\emptyset }\left(Q\left(P-\emptyset \left(P\right)\right)\right)+O_{\in }\left(P\right). \end{array}$$

*Q*(*P*) ∈ *R*^*x*_*n*_^ = state vector ∈(*P*) ∈ *R* = zero mean Gaussian, and *B*, *B*_∅,_*A*, *A*_∅,_ and *O* are identified constant matrices with suitable dimensions. The following condition is satisfied by nonlinear functions *g* and *g*_∅_:

(53) $$\begin{array}{c} \left[g\left(Q\right)-P_{1}Q\right].^{Z}\left[g\left(Q\right)-P_{2}Q\right]\leq 0,{\left[g_{\emptyset }\left(Q\right)-Z_{1}Q\right]}^{Z}\left[g_{\emptyset }\left(Q\right)-Z_{2}Q\right]\leq 0. \end{array}$$

In this, *P*_1_, *P*_2_, *Z*_1_,  and *Z*_2_ are appropriate dimensions real matrices. *P*_1_ = *P*_1_ − *P*_2_ and *Z* = *Z*_1_ − *Z*_2_ are positive symmetric definite matrices. Given filter designed is supposed in this system:

(54) $$\begin{array}{c} \hat{Q}\left(P+1\right)=Y\hat{Q}\left(P\right)+X_{f}\left(P\right). \end{array}$$

To ensure the required security level in the filtering system, an enough condition is derived with stochastic analysis technique. For filter obtaining, inequality linear matrix with constraints of nonlinear is resolved.

### 8.1. Secure Control Approaches of Deception Attack

Deception attack affected discrete time stochastic nonlinear system problem of security control with quadratic cost criterion is studied in [160]. Both actuating and measurement signals were directed to deception as in Figure 11:

(55) $$\begin{array}{c} W\left(P\right)=\hat{W}\left(P\right)+\partial \left(P\right)\in \left(P\right). \end{array}$$

**Figure 12:**
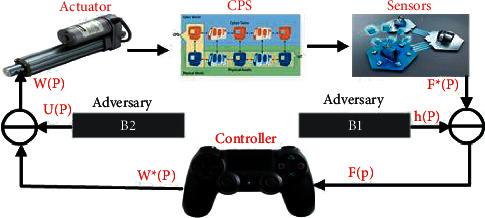
Deception attack schematic.

In Figure 12, B1 and B2 are supposed to be attacker, in system false data system, e.g., $\in \left(P\right)=-\hat{W}\left(P\right)+{ℒ}_{1}\left(P\right)\text{and }h\left(P\right)=-\hat{F}\left(P\right)+{ℒ}_{2}\left(P\right).$,

(56) $$\begin{array}{c} W\left(P\right)=\hat{W}\left(P\right)+\partial \left(P\right)\in \left(P\right), \end{array}$$

(57) $$\begin{array}{c} F\left(P\right)=\hat{F}\left(P\right)+L\left(P\right)h\left(P\right). \end{array}$$

where *W*(*P*) = actuator input, $\hat{W}\left(P\right)$ = controller outputs directed to attacks, *F*(*P*) = controller gained signal, $\hat{F}\left(P\right)$ = sensor measurement directed to attacks, *ϵ*(*P*)and *h*(*P*) = transmitted signals by attacker, and ∂(*P*)and *γ*(*P*) = Bernoulli distributed mutually independent with stochastic variable one and zero, with following probabilities:

(58) $$\begin{array}{c} \text{Prob}\left\{\partial \left(P\right)=1\right\}=\hat{\partial },\\ \text{Prob}\left\{\partial \left(P\right)=0\right\}=1-\hat{\partial }\text{,}\\ \text{Prob}\left\{L\left(P\right)=1\right\}=\hat{L},\\ \text{Prob}\left\{L\left(P\right)=0\right\}=1-\hat{L}. \end{array}$$

In Figure 8, B1 and B2 are supposed to be attacker, in system false data system, e.g., $\in \left(P\right)=-\hat{W}\left(P\right)+{ℒ}_{1}\left(P\right)\text{ and }h\left(P\right)=-\hat{F}\left(P\right)+{ℒ}_{2}\left(P\right)$.

Structuring a dynamic output field or controller feedback is the basic purpose of this delinquent, e.g., given security in possibility is attained while gaining higher limited of the already choose quadratic cost function. Hence, to derive some enough situations by matrix discriminations form in input-to-state framework stability in possibility stochastic analysis approach was pragmatic. To apply matrix inverse lemma controller obtained upper bound.

In [161, 159], there is study of the secure network predictive control system and an architecture for secure and dependent automotive MASs, integrating data message digest algorithm, encryption standard algorithm, predictive control recursive network method, and time-stamp strategy. Predictive control recursive networks rely on time delays, which is pragmatic to ensure the performance of the system, especially when a deception attack influences it.It will accomodate the consequences of attacks and network flaws such as package disorder, package dropout, and time-varying delay.

We studied consensus control and consensus management problem in [162]. There was latest definition of quasi-consensus given for describing the consensus performance with constraints on each agent to keep within few ellipsoidal regions at all-time instant, which based on given topology. In addition, measured result is available for controller from both nearby and individual agents. For gaining quasi-consensus, enough situations are gained with the use of recursive matrix for required control system inequalities.

A resilient control system [139, 163] has been supposed for network control systems effected by false data injections attacks, so that using measurement data and control input they could not be find. Attack of zero variable on plant state variable is not identified during attack, and it seems after result of attack. Hence, a strong Gaussian controller which is linear quadratic is supposed so that there is online updating of Kalman filter from data transferred by an active version of comprehensive prospect ratio detector with the capability to speedy improve of behavior after attack [164].

Actuator attacks and sensor attacks for controller of MASs were proposed in [74, 164–166], and a progressive adaptive strong control scheme is discussed for adversarial mitigating attack in the cyber physical system. Nussbaum function with speedy progressive rate and estimation mechanism is adaptive bound. The double-step back step method was applied to mitigate effects of actuator attack and sensor attacks, and to apply exponentially decaying barrier Lyapunov function, a variable state was controlled.

A feasible control delinquent of MASs data-driven direct to actuator attack class is studied in [166], an unidentified nonstop time linear physical system containing outside instabilities was supposed, and input control signal sent via network layers is supposed to be vulnerable to cyberattacks. For eradicating actuator attack effect, nearby optimal performance and stability of MASs can be gained by data-based adaptive essential sliding-mode control approach. Use of abnormal monitor detection mechanism contains detector threshold information, frequency characteristics, and attack structure for a set of frequency constrained actuator, and sensor attack can be studied in [166]. Explained categorization of cyber threats in cyberattack detection of MASs and secure consensus of MASs has been studied in Table 3.

## 9. Key Challenges

Report [82] tells us clearly that there is no high-level security against upcoming attacks or threats. In addition, an open integrated ecosystem idea for cooperation of security issues was studied. Though, in security system, there should be collaboration of stalk holders, it will have advantage form face threat understanding.

Key challenge for MASs is that there should be no system outside attack, but also from inside, e.g., a worker who is not interested to learn more about the board system. Designing of protective filter based on results of attack measurement for getting high security is one of the key challenges. To see present filtering technologies, those are not sufficient for security assurance, since it is complicated for defenders to get an idea about the time and trick of cyberattack. The Kalman filter method is not enough for MASs, and it is difficult to gain attacker in statically characterization of signal transmitted [107, 171]. In research studies, interest control problem and filtering in concern of security are getting more and attention, e.g., [79] in which there assurance of security against integrity attacks with the use of minimax optimization advancement [167].

With the deficiency of federal reliable power, agent identity verification and creating trust between agents are a big challenge. We can call it decentralization.

Basically, agents use knowledge or information which they get or need from decision-making process environment or another nearby agents. It makes an agent susceptible against malicious entries which may share false data to have effect on agent decision.

Highly important problem is needed to differentiate an accidental failure from an attack. The resulting sign from these accidental situations has chance of similarity, but reaction should not be the same. Fault is repairable. MASs should have capability to defend itself against attacks. Understanding of operations in MASs may be interrupted by malicious attack. A lot of attack stories have been presented in Table 1 of [78].

For MASs, integrity is an important requirement. There is need to pay attention toward sensor networks as well as to data integrity and superstructure. There is also not much more methodology in progressing of secure MAS, so there are several patented results, which may base on possibly exposed approaches.

For designing of applications, we need to see both quality of service and security assurance. For practical applications, considering of multiple attacks is an important point that we can face simultaneously. Present planning of security against several forms of attacks is insufficient for industrialization. In addition, security need and resource constraints as energy limitation and communication bandwidth in practically required to be supposed simultaneously.

Mobile devices are considered threats carrying because these are using several services and external networks. With the progressing of smart wearable mobile applications, loT of challenges presents in progressive of these applications' security measures, as there can be risk of human health and life.

**Figure 13:**
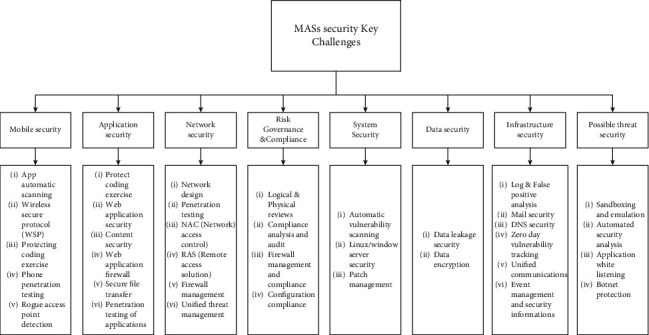
Overall MAS security challenges.

For smart grids, basic challenges are communication protocol weakness, heterogeneity of protocols, and technology and limitation of physical systems.  Table 4 represents research work on MAS challenges.  Table 5 shows summary of different strategies for MAS while Table 6 represents comparison of different methodologies of system security. General security needs in MASs are integrity (which gives surety that since generation, there is no modification in message), authentication (that is sure that each agent is the one claim to be), confidentiality (which gives surety that only allowed agents are able to read specific data), availability, and authorization.  Figure 13 represents overall security challenges in MASs.

## 10. Conclusion and Future Directions

MASs are virtually all around. They can be retrieved and switched remotely, such topographies make susceptible to cyberattacks. There is physical environment process on virtualization and cyber space as a key role for notion plays a central role in MAS. This article explained high-level inclusive discussion regarding various features of MASs that will aid new researchers to cover basic idea of MASs, key challenges in progressing MAS attack, e.g., system failure, virtualization and mobility, and MAS performance methods. First, we studied various attack types in MASs; second, we discussed threats with consistent subtypes and then their possible detection methodologies. After that we give detailed study of MAS attacks and their detection methodologies. Furthermore, an important work of this paper is subjected on several MAS aspects regarding security issues and key challenges. This article will play an important role for researchers to get maximum knowledge about MAS attacks and also to serve as an insightful and overall resources on MASs for researchers.

## Figures and Tables

**Table 1:** Explanation of notations.

Sr. no	Abbreviation	Explanation
1	MAS	Multiagent system
2	MASs	Multiagent systems
3	SN	Sensor network
4	IoT	Internet of Things
5	TCP	Transmission control protocol
6	IP	Internet protocol
7	NICV	Network Interface card virtualization
8	DoS	Denial-of-service
9	PC	Personal computer
10	LTI	Linear time-invariant
11	DDoS	Distributed denial of service
12	MITM	Man-in-the-middle
13	FDI	False data injection
14	PLC	Programmable logic controller
15	SCADA	Supervisory control and data acquisition
16	M2M	Machine to machine
17	SDN	Software define network
18	EECDH	Enhanced Elliptic Curve Differ-Hellman
19	WSN	Wireless sensor network
20	WLS	Weighted least square
21	LQG	Linear-quadratic-Gaussian
22	GT	Game theory

**Table 2:** Types of fault and work on fault estimation, fault detection, and fault tolerant.

Fault type	Fault estimation	Fault detection	Fault tolerant
Actuator fault	[21]	[22]	[23, 24]
Sensor fault	[25, 26]	[27]	[28, 29]
Actuator and sensor fault	[25, 30]	[31, 32]	[19, 33, 34]

**Table 3:** Categorization of cyber threats in cyberattacks, detection, and consensus of MASs.

Attack types	Work on cyberattack detection of MASs	Secure consensus of MASs
Actuator fault	[7, 54,167]	[90]
Sensor fault	[168, 169]	[27]
Actuator and sensor fault	[30, 169]	[31, 170]

**Table 4:** MAS challenges.

Sr. number	Challenges	Research work on MAS challenges
1	Security	[155, 172]
2	Learning	[173, 174]
3	Fault detection	[21, 33, 34, 175]
4	Localization	[176, 177]
5	Task allocation	[178–180]
6	Organization	[176, 177, 181]
7	Formation	[138, 182]
8	Connectivity	[167, 175, 183, 184]
9	Consensus	[167, 175, 183, 184]
10	Control ability	[175, 185, 186]
11	Synchronization	[175, 186]

**Table 5:** Summary of different strategies for MAS.

Application	Goal	Architecture	DRL algorithm	*Q* function estimator	Reference
Maximum power point tracking (MPPT) control	To solve MPPT problem of photovoltaic system in practical condition	Photovoltaic system microgrid base	Deep deterministic policy gradients algorithm	Deep neural network (DNN)	[153]
Secondary control/frequency regulation	With enough load, restoring stability	Hybrid distributed power system as separated small grid	Actor-critic based algorithm	Neural network (NN)	[175]
Bus voltage stability	Designing DC-DC control converter	DC microgrid	Q network/deep *Q* network	NN	[166]

**Table 6:** Comparison of different methodologies of system security.

Benefits	Drawbacks	Methodology
Resiliency to GPS spoofing attack. Speedy and correct finding of anomaly in gained data. Resiliency to GPS spoofing attack	Require a mathematical model of system because there may be complication without any mathematical model when applying to system	Luenberger observer and artificial neural network (ANN) [172]
Resiliency to GPS spoofing and expected attacks	Susceptible to unforeseen attack. Costly against abrupt attack has low accuracy	Machine learning-based algorithm [39]
Low computation load. Resiliency to GPS spoof attack	Susceptible against suspicions and system disturbances, model accuracy dependent complicated to apply on nonlinear system	Model-based detection algorithm [148]
Secure conventional disturbances in between communication connections	Susceptible to GPS spoofing attack	Communication [62, 65]

## Data Availability

The data used to support the findings of this study are available from the corresponding author upon request.
